# A Sensitive and Selective Sensor Based on Orthorhombic Copper Molybdate Decorated on Reduced Graphene Oxide for the Detection of Promethazine Hydrochloride

**DOI:** 10.3390/s25113569

**Published:** 2025-06-05

**Authors:** Venkatachalam Vinothkumar, Yellatur Chandra Sekhar, Shen-Ming Chen, Natesan Manjula, Tae Hyun Kim

**Affiliations:** 1 Department of Chemistry, Soonchunhyang University, Asan 31538, Republic of Korea; sekhar.ycs1@gmail.com; 2 Department of Chemical Engineering and Biotechnology, National Taipei University of Technology, No. 1, Section 3, Chung-Hsiao East Road, Taipei 10608, Taiwan; manjuchemistry93@gmail.com

**Keywords:** Cu_3_Mo_2_O_9_, reduced graphene oxide, antipsychotic drug, human samples, electrochemical sensor

## Abstract

Promethazine hydrochloride (PMH) is a first-generation antipsychotic drug created from phenothiazine derivatives that is widely employed to treat psychiatric disorders in human healthcare systems. However, an overdose or long-term intake of PMH can lead to severe health issues in humans. Hence, establishing a sensitive, accurate, and efficient detection approach to detect PMH in human samples is imperative. In this study, we designed orthorhombic copper molybdate microspheres decorated on reduced graphene oxide (Cu_3_Mo_2_O_9_/RGO) composite via the effective one-pot hydrothermal method. The structural and morphological features of the designed hybrid were studied using various spectroscopic methods. Subsequently, the electrochemical activity of the composite-modified screen-printed carbon electrode (Cu_3_Mo_2_O_9_/RGO/SPCE) was assessed by employing voltammetric methods for PMH sensing. Owing to the uniform composition and structural benefits, the combination of Cu_3_Mo_2_O_9_ and RGO has not only improved electrochemical properties but also enhanced the electron transport between PMH and Cu_3_Mo_2_O_9_/RGO. As a result, the Cu_3_Mo_2_O_9_/RGO/SPCE exhibited a broad linear range of 0.4–420.8 µM with a low limit of detection (LoD) of 0.015 µM, highlighting excellent electrocatalytic performance to PMH. It also demonstrated good cyclic stability, reproducibility, and selectivity in the presence of chlorpromazine and biological and metal compounds. Furthermore, the Cu_3_Mo_2_O_9_/RGO/SPCE sensor displayed satisfactory recoveries for real-time monitoring of PMH in human urine and serum samples. This study delivers a promising electrochemical sensor for the efficient analysis of antipsychotic drug molecules.

## 1. Introduction

Promethazine hydrochloride (PMH), chemically known as N, N-dimethyl-1-phenothiazin-10-yl-propan-2-amine hydrochloride, is a first-generation phenothiazine derivative extensively utilized for its sedative, analgesic, anticholinergic, antihistaminic, and antipsychotic effects [1]. It is also effective at relieving symptoms like vomiting and nausea, motion sickness, and allergic diseases in human beings [2]. Nevertheless, excess or high-level PMH use may have adverse effects on human health, such as endocrine variations, cardiac changes, reproductive dysfunction, and occasional hypotension [3]. Moreover, European and American countries have forbidden the consumption of phenothiazine drugs because of high rates of usage among teenagers and young adults [4]. Therefore, designing a sensitive, reliable, and precise detection technique is vital to PMH monitoring in human samples.

Numerous approaches have been deployed thus far to identify and determine PMH, such as capillary zone electrophoresis [5], chemiluminescence [6], spectrofluorometry [7], colorimetry [8], turbidimetry [9], and high-performance liquid chromatography [10]. However, the above-listed techniques are pricey, have complex protocols, take a long time, and require trained operators. The electroanalytical technique features significant benefits over other approaches, which include cost-efficiency, high sensitivity, simple operation, high precision, short-term field analysis, and portability [2,11]. Screen-printed carbon electrodes (SPCEs) are currently utilized to design innovative sensing platforms and improve performance due to their low cost, ease of access to usage, better reproducibility, compatibility, disposability, and quick large-scale fabrication [12]. Additionally, the surface of SPCEs is adjustable and easily altered with chemical substances, thus making it suitable for monitoring various electrochemical sensors and biosensors [13]. Considering developmental aims, the electrochemical technique was applied to detect PMH analyte using the modified SPCEs.

Transition bimetal oxides (TBOs) have recently drawn much interest in the development of electrochemical sensors and biosensors because of their uniform shape, unique size, tailored structure, even composition, highly active sites, and better electrical conductivity [14,15]. Transition metal molybdates, such as CoMoO_4_, NiMoO_4_, and Cu_3_Mo_2_O_9_, are considered potential electrodes for various sensors [15,16], and energy storage [17,18] applications thanks to their cost-effective, eco-friendly, multi-oxidation states, good chemical stability, and strong electrocatalytic activity. As a crucial component of metal molybdate, copper molybdate (Cu_3_Mo_2_O_9_) is a significant p-type semiconductor with a low band gap of 2.1 eV [19]. Owing to its abundance, biocompatibility, nontoxic, stable structure, redox nature, environmental friendliness, high theoretical capacity, and improved electrochemical properties, it is of great interest in the fields of luminescence, photocatalysts, and electrocatalysts [19,20]. Moreover, the orthorhombic composition of Cu_3_Mo_2_O_9_ with the Pnma space group has active sites that promote a rapid electrochemical response for the analyte [18]. Nonetheless, low sensitivity and poor selectivity are typical problems for electrochemical sensors. As a result, adding or doping a foreign element into Cu_3_Mo_2_O_9_ is an excellent choice for improving its physicochemical characteristics.

Carbon and its derivative materials possess good mechanical strength, high mobility, large surface area, and thin structure [2]. Reduced graphene oxide (RGO) is regarded as a potential material for electrochemical sensors due to its active functional groups, better electrical conductivity, and high chemical stability [21]. Further, RGO and TBOs have synergistic effects, improving electron transfer to drug detection. Also, TBOs combined with RGO to develop promising composite-based sensor electrode materials that are highly sensitive, reliable, and precise [14]. For instance, our group recently developed TBOs anchored on RGO for the electrochemical monitoring of sunset yellow, and it exhibited superior electrocatalytic activity [21]. In addition, S. M. Ghoreishian et al. proposed a heterostructured Cu_3_Mo_2_O_9_/RGO/g-C_3_N_4_ composite with enhanced photoelectrochemical and photodegradation activity [20]. To the best of our knowledge, the introduction of a Cu_3_Mo_2_O_9_/RGO hybrid for electrochemical detection has not been explored. In light of this, a simple and effective method of concurrently growing Cu_3_Mo_2_O_9_ and reducing graphene oxide was developed for the one-step hydrothermal process of Cu_3_Mo_2_O_9_ on the surface of RGO sheets. This one-step route of Cu_3_Mo_2_O_9_ on RGO is a promising strategy to make a combined composite catalyst to boost the electrochemical sensor performance.

Based on the above considerations, this study developed a sensitive modified electrode based on a Cu_3_Mo_2_O_9_/RGO hybrid to determine PMH for the first time via a one-step hydrothermal technique. The crystalline structure, functional groups, chemical composition, surface morphology, and elemental proportions of the designed composite were examined by diverse analytical techniques, including XRD, FTIR, Raman, XPS, FESEM, and EDX instruments. Their electrochemical attributes were analyzed using EIS, CV, and DPV profiles. Because of the high conductivity of RGO and the high crystallinity of Cu_3_Mo_2_O_9_, the as-fabricated Cu_3_Mo_2_O_9_/RGO/SPCE revealed improved electrochemical activity. The electron transfer rate of the composite electrode towards PMH was further boosted by a synergistic effect. Consequently, the Cu_3_Mo_2_O_9_/RGO/SPCE demonstrated broad linearity and a lower detection limit than the previous PMH sensors encompassed in our comparison table. Furthermore, this sensor proved high selectivity, better reproducibility, and satisfactory cyclic stability to PMH. The practicability of PMH on Cu_3_Mo_2_O_9_/RGO/SPCE in human samples was tested through the DPV method, and good recoveries were found.

## 2. Materials and Methods

### 2.1. Materials

All materials used throughout this study were analytical-grade and without purification. Copper (II) nitrate trihydrate (Cu(NO_3_)_2_·3H_2_O), ammonium heptamolybdate tetrahydrate ((NH_4_)_6_Mo_7_O_24_·4H_2_O), urea (CH_4_N_2_O), potassium chloride (KCl), potassium permanganate (KMnO_4_), sodium nitrate (NaNO_3_), sodium hydroxide (NaOH), hydrochloric acid (HCl), sulfuric acid (H_2_SO_4_), ethanol (C_2_H_5_OH), monosodium dihydrogen phosphate (NaH_2_PO_4_), disodium hydrogen phosphate (Na_2_HPO_4_), graphite powder, and PMH were purchased to Sigma Aldrich. The screen-printed carbon electrodes (SPCE) were purchased from Zensor R&D Co., Ltd. (Taichung City, Taiwan). The 0.1 M phosphate buffer solution (PBS) was prepared by mixing NaH_2_PO_4_ and Na_2_HPO_4_ in distilled water. The different pH solutions were adjusted with either liquid HCl or NaOH.

### 2.2. Synthesis of Cu_3_Mo_2_O_9_/RGO Hybrid

**Scheme 1:**
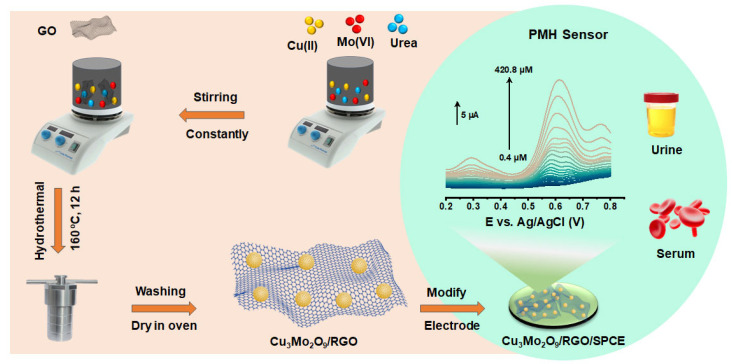
The preparation and fabrication of Cu_3_Mo_2_O_9_/RGO/SPCE toward PMH detection.

Graphene oxide (GO) was synthesized using a modified Hummers method, as detailed in our previous report [11]. The hybrid of Cu_3_Mo_2_O_9_/RGO was synthesized through a one-pot hydrothermal technique. Typically, 0.1 M of Cu(NO_3_)_2_·3H_2_O and 0.1 M of (NH_4_)_6_Mo_7_O_24_·4H_2_O were dissolved in 60 mL of distilled water with continuous stirring, followed by 0.3 M of CH_4_N_2_O addition. After achieving a homogeneous solution, 5 mg of GO was added to the above solution and constantly stirred for 20 min at ambient temperature. The mixed suspension was then transferred into a 100 mL Teflon-lined stainless steel autoclave and heated at 160 °C for 12 h in an oven. The black mixture was naturally cooled at room temperature and washed many times with distilled water and ethanol via centrifuging. Finally, the black precipitate was dried at 60 °C overnight to obtain the Cu_3_Mo_2_O_9_/RGO hybrid. For comparison, the pure Cu_3_Mo_2_O_9_ microspheres were developed by the above procedure without the GO addition. The preparation and fabrication of Cu_3_Mo_2_O_9_/RGO/SPCE for the PMH sensor is illustrated in Scheme 1.

### 2.3. Instrumentation

The crystallinity and phase identity of all samples were studied through an X-ray diffractometer (XRD) with the Panalytical X’PERT PRO analyzer (Almelo, The Netherlands) equipped with a Cu Kα radiation source (λ = 1.5406 Å) operated at 40 kV and 30 mA. XRD profiles were measured over a 2θ range of 10–70° with a step size of 0.02° and scan rate of 0.5° min^−1^. The chemical functionality of the as-proposed samples was evaluated via Fourier transform infrared spectroscopy (FT-IR) using JASCO 4600LE equipment (Tokyo, Japan) in the range of 400–4000 cm^−1^ with a number of scans of 12 and a resolution of 4 cm^−1^. Raman active bands of as-prepared materials were recorded on the UniBioTech-ACORN (Seoul, Republic of Korea) spectrometer with a laser excitation of 532 nm. X-ray photoelectron spectroscopy (XPS) determined the oxidation states in the composite using the Thermo Fisher Scientific Multi-Lab 2000 equipped (Waltham, MA, USA) in monochromic Al Kα (hν = 1486.6 eV) X-ray source with a sampling area of 400 µm diameter, resolution of 0.7 eV, X-ray power of 12 kV, 3 mA, step size of 1 eV, and scan pass energy of −200 eV. The morphological and elemental studies of Cu_3_Mo_2_O_9_ and Cu_3_Mo_2_O_9_/RGO hybrid were analyzed by a field emission scanning electron microscope (FESEM) JEOL-JSM-6500F model (JEOL Ltd., Peabody, MA, USA) operated at an accelerating voltage of 5–15 kV. The electrochemical tests, including electrochemical impedance spectroscopy (EIS), cyclic voltammetry (CV), and differential pulse voltammetry (DPV) were executed in a three-electrode arrangement utilizing XPot-ZAHNER-Elektrik and CHI410A instruments (CHI Instruments, Inc., Bee Cave, TX, USA). The bare or modified screen-printed carbon electrode (SPCE) is the working electrode, whilst the counter and reference electrodes were the Pt wire and saturated Ag/AgCl. EIS measurements were conducted in the frequency range of 0.1 Hz to 10^5^ kHz using a 5 mV amplitude potential.

### 2.4. Fabrication of Cu_3_Mo_2_O_9_/RGO/SPCE

Prior to catalyst modification, the SPCE surface was gently rinsed with deionized water and dried at room temperature. On the other hand, for fabricating Cu_3_Mo_2_O_9_/RGO/SPCE, the Cu_3_Mo_2_O_9_/RGO hybrid (5 mg) was sonicated with distilled water (1 mL) for 20 min. After obtaining a uniform suspension, the SPCE was coated with Cu_3_Mo_2_O_9_/RGO hybrid (8 µL) and then allowed to dry in an oven for 10 min at 60 °C. Finally, the designed Cu_3_Mo_2_O_9_/RGO/SPCE was applied to electrochemical PMH monitoring in optimal conditions. The same fabrication process was followed to fabricate individual Cu_3_Mo_2_O_9_/SPCE and RGO/SPCE for PMH detection.

### 2.5. Preparation of Real Samples

Human samples were employed to assess the reliability of the Cu_3_Mo_2_O_9_/RGO/SPCE sensor in practical applications. Samples of human urine and serum were collected from healthy volunteers at Chang-Gung Memorial Hospital. Both samples were centrifuged for 20 min at 8000 rpm, followed by filtration through Whatman (1–55 mm diameter) filter paper. The sample supernatant was then diluted with 0.1 M PBS (pH 7) solution. Subsequently, known PMH concentrations were injected into the diluted real samples. Lastly, the prepared samples were tested to PMH via the DPV technique.

## 3. Results and Discussion

### 3.1. Physical Characterization of Cu_3_Mo_2_O_9_/RGO Hybrid

**Figure 1:**
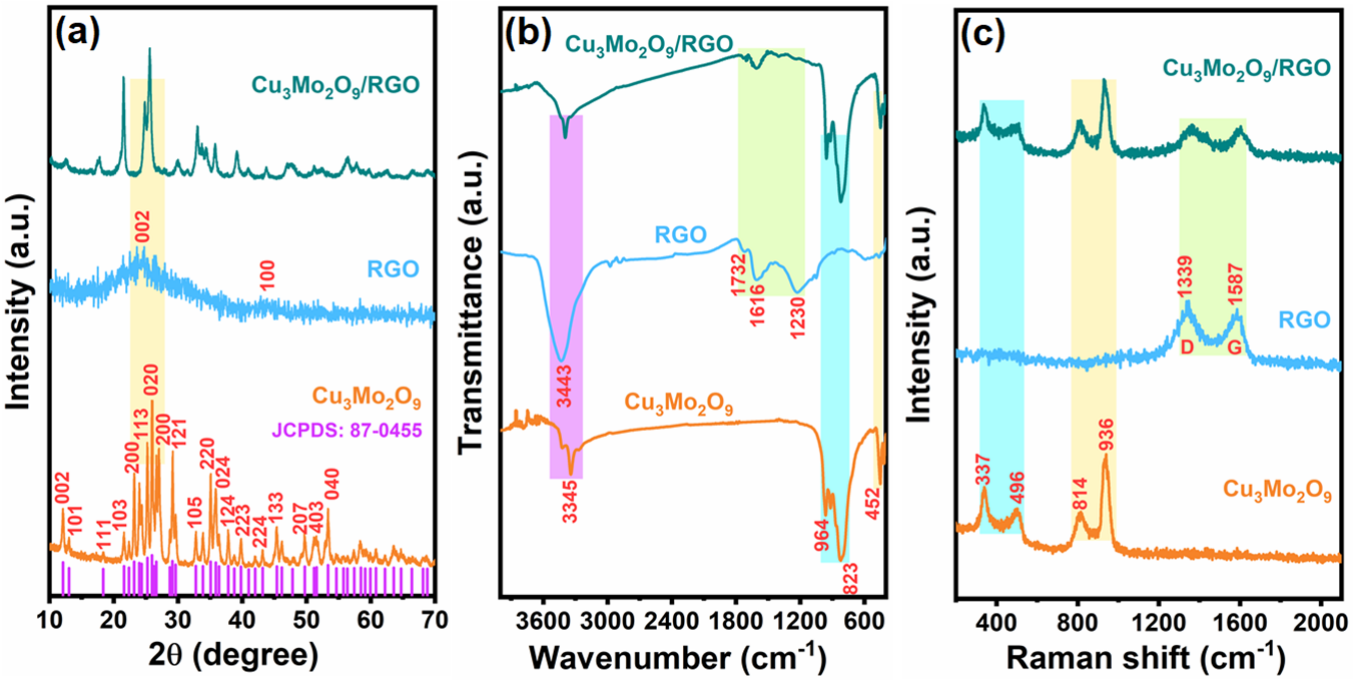
(**a**) XRD patterns, (**b**) FTIR spectrums, and (**c**) Raman bands for Cu_3_Mo_2_O_9_, RGO, and Cu_3_Mo_2_O_9_/RGO hybrid.

The crystalline structure, phase purity, and composition of Cu_3_Mo_2_O_9_, RGO, and Cu_3_Mo_2_O_9_/RGO electrocatalysts were analyzed through an X-ray diffractometer (XRD) between 10° and 70°. As displayed in Figure 1a, XRD peaks of Cu_3_Mo_2_O_9_ were observed for 2θ values at 12.1°, 13.03°, 18.36°, 21.60°, 22.37°, 23.18°, 23.98°, 24.35°, 25.23°, 25.94°, 26.23°, 26.61°, 28.72°, 29.13°, 29.63°, 32.77°, 33.87°, 35.07°, 35.88°, 36.38°, 37.81°, 38.77°, 39.79°, 41.02°, 42°, 43.17°, 45.38°, 46.17°, 47.82°, 49.74°, 51.17°, 51.57°, 53.36°, 54.65°, 55.75°, 56.36°, 57.44°, 58.46°, 59.15°, 59.95°, 60.81°, 62.22°, 63.60°, 64.72°, 66.41°, 68.14°, and 68.82° correspond to the (002), (101), (111), (103), (013), (200), (201), (004), (113), (020), (202), (210), (022), (121), (203), (105), (204), (220), (024), (214), (124), (205), (223), (215), (313), (224), (133), (230), (401), (207), (403), (217), (040), (325), (208), (422), (218), (128), (415), (037), (144), (243), (327), (432), (433), (522), and (328) crystal planes, respectively. These planes all coincide with the orthorhombic structure of Cu_3_Mo_2_O_9_ (JCPDS: 87-0455) [20], suggesting the purity of the as-synthesized sample. Moreover, the broader peaks at around 25° and 45° relate to (002) and (100) planes, revealing the presence of RGO [21]. In the Cu_3_Mo_2_O_9_/RGO hybrid, Cu_3_Mo_2_O_9_ peaks diminished due to the close contact between the two materials [20]. Also, the RGO peaks are not visible because of the lower composition and inferior crystallinity of RGO in the Cu_3_Mo_2_O_9_/RGO. However, the high-intensity peaks of Cu_3_Mo_2_O_9_ and low-intensity peaks of RGO present in the composite with a smaller shift in the 2θ, supporting the construction of the Cu_3_Mo_2_O_9_/RGO hybrid.

For analyzing the vibrational properties of as-prepared electrocatalysts, FTIR and Raman spectroscopy are crucial analytical techniques. The FTIR spectra of Cu_3_Mo_2_O_9_, RGO, and Cu_3_Mo_2_O_9_/RGO are shown in Figure 1b. The main FTIR bands of Cu_3_Mo_2_O_9_ at 452 cm^−1^ and 823 cm^−1^ reflect the stretching vibrations of Cu–O and Mo–O bands [22]. Also, the 964 cm^−1^ peak corresponds to the v1 vibration of the deformed MoO_4_ tetrahedral [20], whereas the 3345 cm^−1^ broad peak relates to the absorbed water molecules of -OH stretching [23]. The standard RGO bands were identified at 1230 cm^−1^, 1616 cm^−1^, 1732 cm^−1^, and 3443 cm^−1^ corresponding to the stretching modes of C–OH, C=C, C=O, and O–H, respectively [24]. While for Cu_3_Mo_2_O_9_/RGO, the characteristic peaks of Cu_3_Mo_2_O_9_ and RGO were reassembled, further endorsing the Cu_3_Mo_2_O_9_/RGO hybrid. Figure 1c exhibits the Raman bands of Cu_3_Mo_2_O_9_, RGO, and Cu_3_Mo_2_O_9_/RGO. The Raman active modes of Cu_3_Mo_2_O_9_ are revealed in four bands at 337 cm^−1^, 496 cm^−1^, 814 cm^−1^, and 936 cm^−1^, respectively. The bands at 337 cm^−1^ and 496 cm^−1^ denote the existence of Cu–O in Cu_3_Mo_2_O_9_ [25]. On the other hand, the 814 cm^−1^ band is allocated to the interconnected Mo–O–Mo stretching mode, whilst the 936 cm^−1^ band is assigned to the Mo–O symmetric stretching mode [26]. In the case of RGO, the D and G bands originated at 1339 cm^−1^ and 1587 cm^−1^, which are indicative of the disorder of graphene and graphitic carbon [24]. The intensity ratios (I_D_/I_G_) for RGO and Cu_3_Mo_2_O_9_/RGO are 0.843 and 0.851, suggesting the addition of Cu_3_Mo_2_O_9_ to RGO induces a boost in defects and vacancies in Cu_3_Mo_2_O_9_/RGO. Remarkably, the detected peaks of two materials (Cu_3_Mo_2_O_9_ and RGO) reemerged in the hybrid, evidencing the integration of Cu_3_Mo_2_O_9_/RGO. As a result, the FTIR and Raman analyses of the developed Cu_3_Mo_2_O_9_/RGO showed effective structural confirmation.

**Figure 2:**
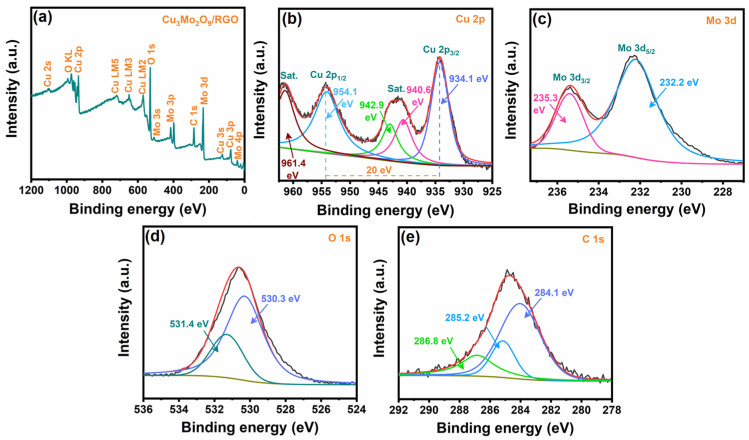
(**a**) XPS full survey scan of Cu_3_Mo_2_O_9_/RGO, and the core level spectrum for (**b**) Cu 2p, (**c**) Mo 3d, (**d**) O 1s, and (**e**) C 1s.

The elemental configuration, surface atomic composition, and chemical states of Cu_3_Mo_2_O_9_/RGO were studied via XPS (Figure 2), and the red and black lines in subfigures (Figure 2b–e) indicate the fitted data and raw data of the composite. As illustrated in Figure 2a, the full survey scan of the Cu_3_Mo_2_O_9_/RGO hybrid disclosed only the Cu, Mo, O, and C atoms, with no impurities. In the Cu 2p high-resolution XPS spectrum (Figure 2b), two binding energy peaks at 934.1 eV and 954.1 eV match Cu 2p_3/2_ and Cu 2p_1/2_ [17], and their satellite (Sat.) peaks are 940.6 eV, 942.9 eV, and 961.4 eV, respectively. The peak-to-peak separation between Cu 2p_3/2_ and Cu 2p_1/2_ was 20 eV [27], implying that Cu is present in the +2 valence state in Cu_3_Mo_2_O_9_/RGO. The Mo 3d spectra in Figure 2c display two distinct peaks at binding energies of 232.2 eV and 235.3 eV, representing Mo 3d_5/2_ and Mo 3d_3/2_. These peaks confirm the occurrence of Mo^6+^ in the hybrid [22]. Furthermore, the O 1s deconvoluted peaks at 530.3 eV and 531.4 eV indicated the metal-to-oxygen (M–O) and hydroxyl oxygen (O–H) species [17,20] (Figure 2d). The C 1s (Figure 2e) revealed three RGO peaks in Cu_3_Mo_2_O_9_/RGO: C–C/C=C (284.1 eV), C–O (285.2 eV), and C=O (286.8 eV) [28,29]. These XPS spectra validated the amalgamation of Cu_3_Mo_2_O_9_ and RGO, tallying with the structural and vibrational properties.

**Figure 3:**
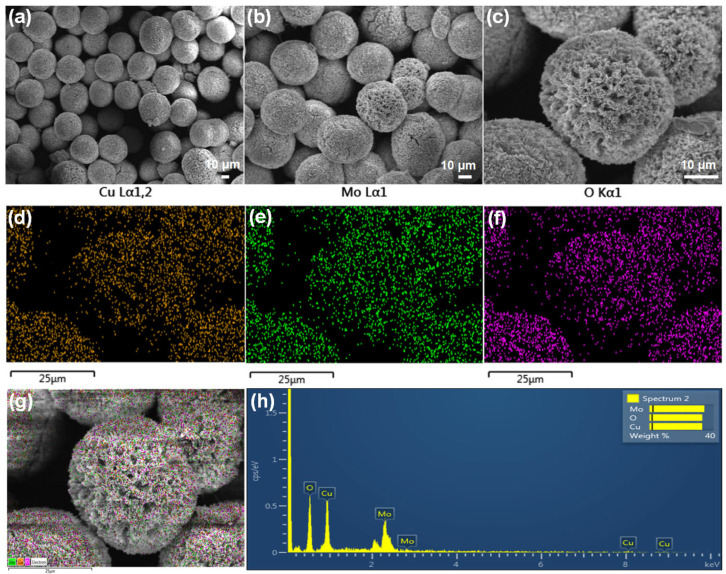
(**a**–**c**) FESEM images, (**d**–**g**) elemental mapping, and (**h**) EDX spectrum for pure Cu_3_Mo_2_O_9_.

The unified formation of Cu_3_Mo_2_O_9_ on the surface of RGO is necessary to achieve the best electrochemical properties. The chemical compositions, surface morphologies, and elemental proportions of as-synthesized Cu_3_Mo_2_O_9_ and Cu_3_Mo_2_O_9_/RGO were investigated by the FESEM technique. As indicated in Figure 3a–c, the FESEM images of Cu_3_Mo_2_O_9_ showcase the homogeneous three-dimensional (3D) sphere structure with a micro-grade size. These uniform microspheres possess a porous structure having vacant spaces and active sites, which facilitate charge transmission at the electrolyte and electrode interface. The elemental mapping and EDX results of Cu_3_Mo_2_O_9_ in Figure 3d–h demonstrate that the chemical components, including Cu, Mo, and O were distributed evenly throughout the microspheres with atomic weights of 32.89%, 34.20%, and 32.91%.

**Figure 4:**
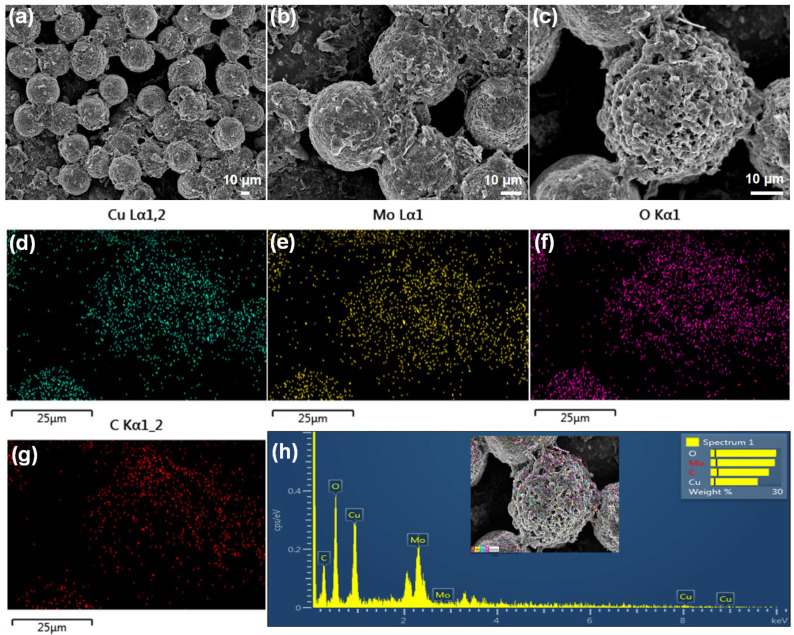
(**a**–**c**) FESEM images, (**d**–**g**) elemental mapping, and (**h**) EDX spectrum of hybrid Cu_3_Mo_2_O_9_/RGO.

Moreover, Figure 4a–c depicts the FESEM images of Cu_3_Mo_2_O_9_/RGO, revealing that the 3D Cu_3_Mo_2_O_9_ microspheres are decorated on RGO sheets. This suggests that efficient interaction between Cu_3_Mo_2_O_9_ and RGO was achieved using the one-pot hydrothermal approach. The integrated Cu_3_Mo_2_O_9_/RGO significantly improves conductivity and surface area, allowing rapid electrochemical reactions. In addition, the uniformly composed Cu_3_Mo_2_O_9_/RGO accelerates electron transport and cycle stability by increasing the electrode-material contact surface. The elemental mapping illustrates that the atoms of Cu, Mo, O, and C were uniformly distributed in the hybrid (Figure 4d–g). The EDX spectrum further verifies the existence of all elements in Cu_3_Mo_2_O_9_/RGO with atomic weights of 20.02%, 27.31%, 27.87%, and 24.80%, respectively (Figure 4h). These findings indicate the successful fabrication and purity of the Cu_3_Mo_2_O_9_/RGO hybrid.

### 3.2. Electrochemical Impedance Analysis at Different Modified Electrodes

**Figure 5:**
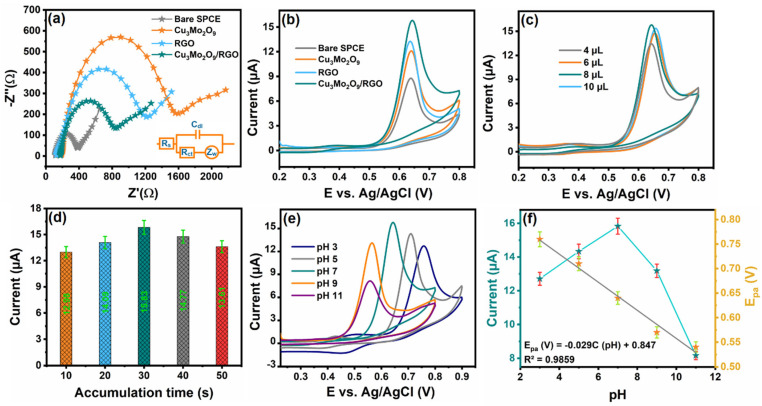
(**a**) EIS-Nyquist curves of bare SPCE, Cu_3_Mo_2_O_9_/SPCE, RGO/SPCE, and Cu_3_Mo_2_O_9_/RGO/SPCE in 0.1 M KCl and 5 mM [Fe(CN)_6_]^3–/4–^, (**b**) CV profiles of PMH on various interfaces containing 0.1 M PBS (pH 7) at 50 mV s^−1^, (**c**) CV response for various loading catalyst, (**d**) CV response of Cu_3_Mo_2_O_9_/RGO/SPCE with different accumulations to PMH, (**e**) CV curves of modified electrocatalyst in 50 µM PMH under various pH values, and (**f**) calibration plot representing current and potential versus pH.

Electrochemical impedance spectroscopy (EIS) was employed to assess the charge transfer resistance (R_ct_) characteristics for various modified and bare electrodes in 0.1 M KCl and 5 mM [Fe(CN)_6_]^3–/4–^. The Nyquist plot has two parts: the semicircle at high frequencies represents the R_ct_, whereas the linear section at low frequencies reflects the diffusion process. EIS experiments were undertaken at frequencies ranging from 0.1 Hz to 10^5^ kHz and a working potential of 5 mV using the CHI410A instrument. Figure 5a depicts the EIS curves for bare SPCE, Cu_3_Mo_2_O_9_/SPCE, RGO/SPCE, and Cu_3_Mo_2_O_9_/RGO/SPCE, along with the Randles circuit (inset of Figure 5a). The R_ct_ at the bare SPCE was 269 Ω, due to the electrochemical properties of the electrode and [Fe(CN)_6_]^3–/4–^ solution [30]. After the SPCE was modified with as-proposed materials, the R_ct_ for Cu_3_Mo_2_O_9_/SPCE, RGO/SPCE, and Cu_3_Mo_2_O_9_/RGO/SPCE was 1401 Ω, 1089 Ω, and 663 Ω, respectively. Notably, the Cu_3_Mo_2_O_9_/RGO/SPCE revealed the lowest R_ct_ (663 Ω) compared to other modifiers (Cu_3_Mo_2_O_9_/SPCE, RGO/SPCE), implying that the charge transfer rate was greatly enhanced on the Cu_3_Mo_2_O_9_/RGO hybrid. Furthermore, owing to the synergistic effect and electrostatic repulsion between the electrolyte and the electrode surface, a significant decrease in R_ct_ (663 Ω) of Cu_3_Mo_2_O_9_/RGO/SPCE improves electrical conductivity, thereby boosting better electrochemical activity towards PMH detection.

### 3.3. Electrochemical Behavior of PMH on Various Interfaces

The electrochemical activities of PMH were examined by CV at various interfaces, including bare SPCE, Cu_3_Mo_2_O_9_/SPCE, RGO/SPCE, and Cu_3_Mo_2_O_9_/RGO/SPCE with the addition of 50 µM PMH in 0.1 M PBS (pH 7). The effect of PMH oxidation current response on different electrodes is displayed in Figure 5b, wherein the bare SPCE exhibited a low oxidation peak (8.78 µA). However, when the Cu_3_Mo_2_O_9_ and RGO were constructed on SPCEs, the oxidation peak was improved to 12.14 µA and 13.32 µA, respectively, relative to the bare electrode because Cu_3_Mo_2_O_9_ and RGO have good structural properties, which promote the electrochemical reaction for PMH. Conversely, the designed Cu_3_Mo_2_O_9_/RGO/SPCE showcased a higher peak current of 15.83 µA at 0.641 V than the individual modified (Cu_3_Mo_2_O_9_/SPCE and RGO/SPCE) and unmodified electrodes. The better electrooxidation performance of Cu_3_Mo_2_O_9_/RGO/SPCE not only improves the surface attributes but also increases the conductivity, accelerating electron transfer to PMH. Furthermore, the Cu_3_Mo_2_O_9_/RGO/SPCE was 1.80 times greater than bare SPCE due to the synergistic interaction of Cu_3_Mo_2_O_9_ and RGO, which enhances the PMH electrochemical activity.

### 3.4. Experimental Condition Optimization

Several parameters were optimized to enhance the sensitivity of Cu_3_Mo_2_O_9_/RGO/SPCE, including the loading catalyst, the accumulation time, and the pH of the buffer solution.

#### 3.4.1. Influence of Loading Catalyst

The influence of the loading catalyst on PMH was studied in 0.1 M PBS (pH 7) at 50 mV s^−1^. Figure 5c portrays the various loading amounts of 4 µL, 6 µL, 8 µL, and 10 µL of Cu_3_Mo_2_O_9_/RGO/SPCE. The highest oxidation current appears at 8 µL loading, indicating the electrochemical performance of PMH is the best at this time. When the loading amount goes above 8 µL, the oxidation current reduces due to the thicker electrode coating that hinders electron transfer to PMH. Hence, 8 µL of Cu_3_Mo_2_O_9_/RGO/SPCE was employed to carry out all PMH studies.

#### 3.4.2. Influence of Accumulation Time

Figure 5d exhibits the influence of varying accumulation durations (10–50 s) of Cu_3_Mo_2_O_9_/RGO/SPCE for PMH using the CV technique. When the accumulation time increased from 10 s to 30 s, a gradual increase in the oxidation current was observed, indicating improved adsorption of PMH on the electrode surface. Beyond 30 s, the oxidation current slightly decreased, due to the saturation of the electrode surface. Therefore, 30 s was selected as the optimal accumulation time for subsequent experiments.

#### 3.4.3. Influence of pH

The electrolyte pH substantially influences the PMH performance at Cu_3_Mo_2_O_9_/RGO/SPCE. Figure 5e provides the CV profiles of PMH at different electrolyte pH levels (3 to 11). In this analysis, the oxidation current of PMH increased gradually from pH 3 to 7, and thereafter the oxidation response diminished as pH increased (7 to 11). Accordingly, the highest oxidation current was recorded at pH 7. Further, the electrooxidation potential of the PMH moved to the negative side as pH grew, which demonstrates that protons participate in the electrochemical oxidation of PMH [2,31]. Figure 5f presents a linear plot of pH values against oxidation current and oxidation peak potential, following the regression equation E_pa_ (V) = −0.029C (pH) + 0.847 (R^2^ = 0.9859). This confirms that the electrolyte pH 7 is ideal for PMH monitoring at Cu_3_Mo_2_O_9_/RGO/SPCE, and it is utilized throughout all electrochemical investigations.

### 3.5. Influence of Increasing Concentration and Scan Rate

**Figure 6:**
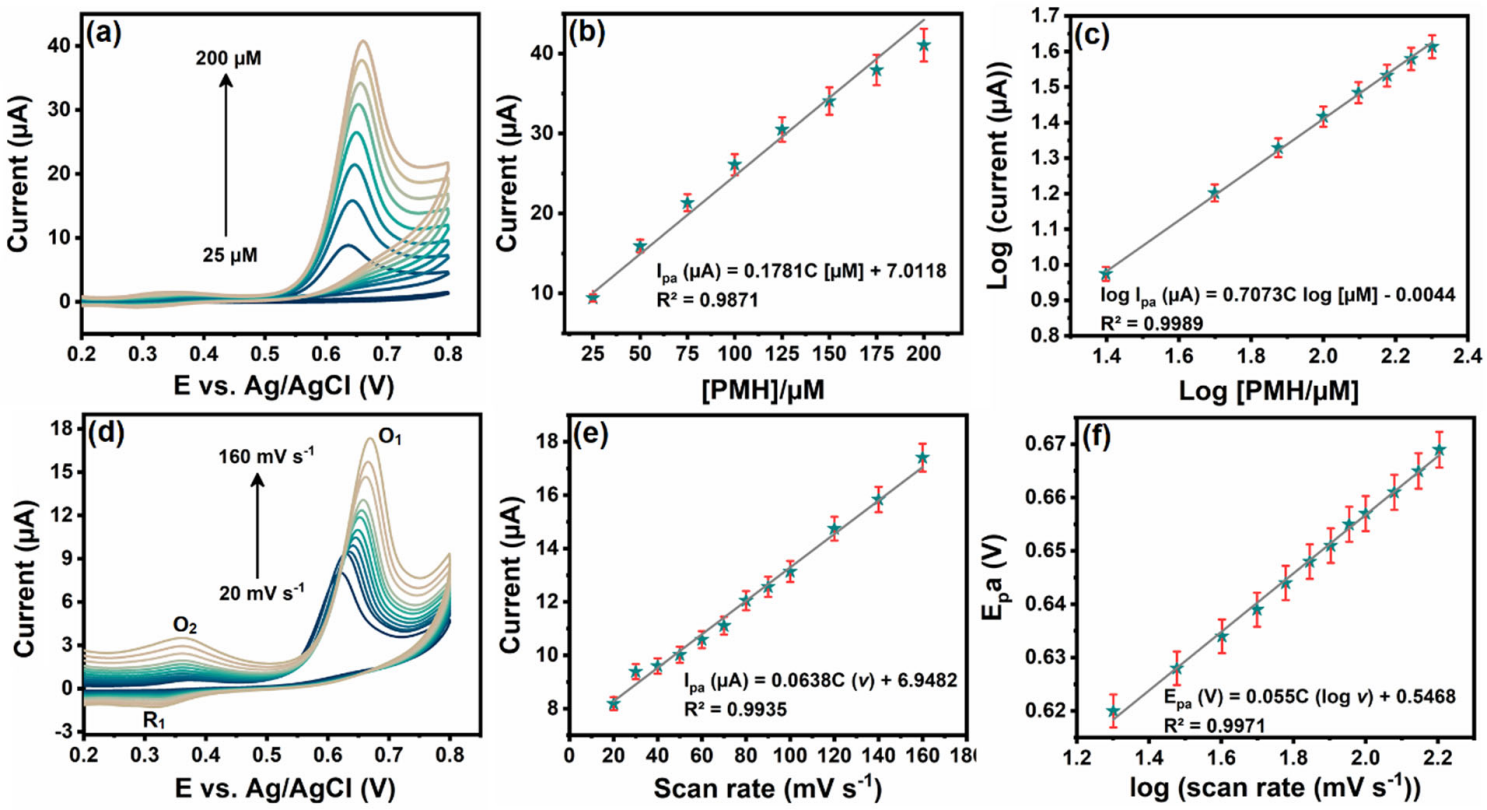
(**a**) CV of Cu_3_Mo_2_O_9_/RGO/SPCE in varying concentrations of PMH, (**b**) linear plots of PMH additions versus current, and (**c**) log concentrations of PMH versus log current. (**d**) CV recorded different scan sweeps for Cu_3_Mo_2_O_9_/RGO/SPCE, (**e**) calibration curves of oxidation current versus scanning rates, and (**f**) oxidation potential versus log scanning rates.

To determine the influence of analyte concentration on the electrochemical system, CV was performed at different PMH concentrations (25–200 µM) using Cu_3_Mo_2_O_9_/RGO/SPCE in a pH 7 solution. As concentration increased from 25 to 200 µM (Figure 6a), the CV of the PMH oxidation current improved linearly. Figure 6b shows a linear regression equation for peak current versus PMH concentration: I_pa_ (µA) = 0.1781C [µM] + 7.0118 (R^2^ = 0.9871). In addition, the linear plot of log peak current against log PMH concentration with regression equation of log I_pa_ (µA) = 0.7073C log [µM] − 0.0044 (R^2^ = 0.9989) (Figure 6c), authenticates that Cu_3_Mo_2_O_9_/RGO/SPCE had excellent electrocatalytic activity for PMH detection. Because of the abundance of active sites on Cu_3_Mo_2_O_9_/RGO, which rapidly absorb all concentrations of PMH and may subsequently be highly oxidized. Thus, we found that the Cu_3_Mo_2_O_9_/RGO hybrid is an efficient electrode material for the sensitive and selective determination of PMH.

To gain insight into the reaction kinetics and mechanism for PMH on Cu_3_Mo_2_O_9_/RGO/SPCE, CV profiles were analyzed at scan rates ranging from 20 to 160 mV s^−1^. As shown in Figure 6d, the oxidation followed by reduction peaks of PMH increases steadily as the scanning rates rise to 20–160 mV s^−1^. Their peak potential shifts towards more positive values, due to the time needed to finish a full cycle under the influence of the scan rate. Also, the oxidation current is directly proportional to the scan sweeps, evidenced by Figure 6e, with the linear regression equation I_pa_ (µA) = 0.0638C (v) + 6.9482 (R^2^ = 0.9935). According to these results, the electrochemical mechanism of PMH at the Cu_3_Mo_2_O_9_/RGO/SPCE sensor was controlled by the typical adsorption process [1,2]. Moreover, the number of PMH electrons (n) involved in the Cu_3_Mo_2_O_9_/RGO/SPCE was determined on the slope of the calibration plot of E_pa_ versus log v (Figure 6f), and the linear regression equation E_pa_ (V) = 0.055C (log v) + 0.5468 (R^2^ = 0.9971). Using Laviron’s formula [30]: $E_{pa}\left(V\right)=E^{0}+\frac{2.303RT}{\propto nF}log\frac{RTk^{0}}{\propto nF}+\frac{2.303RT}{\propto nF}\log v$, where R, T, F, α, E^0^, and k^0^ denote the gas constant (8.314 J mol^−1^ K^−1^), temperature (293.15 K), Faraday’s constant (96,480 C mol^−1^), electron transfer coefficient (considered to be 0.5), formal potential, and heterogeneous rate constant. Therefore, the n for PMH that participated in the rate-determining step was estimated to be 2.11 (=2). Consequently, the Cu_3_Mo_2_O_9_/RGO/SPCE sensor contributed to two electron transfer toward PMH, which agrees with previously modified sensors [32,33,34].

**Scheme 2:**
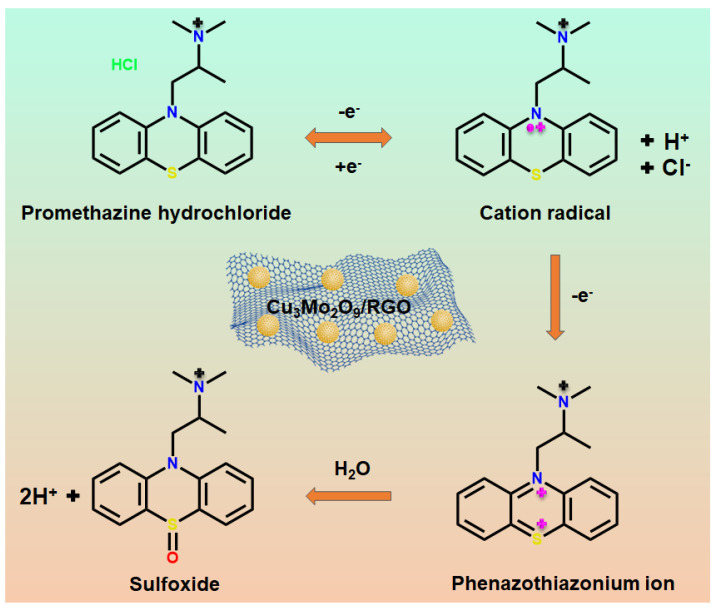
The proposed electrochemical mechanism for PMH on Cu_3_Mo_2_O_9_/RGO/SPCE.

Scheme 2 portrays the probable electrochemical mechanism for PMH. It involves a two-electron transfer. The first electron loss from PMH to form the cation radical represents the maximum oxidation peak (O_1_) at 0.641 V. Following this, the second electron loss to form the phenazothiazonium ion indicates the small reduction peak (R_1_) at about 0.32 V. Further, the second oxidation peak (O_2_) was seen at about 0.35 V in the reverse scan, originating from mono or di-hydroxylated forms of PMH, consistent with earlier PMH reports [35,36]. It is worth noting that Cu_3_Mo_2_O_9_/RGO/SPCE shows the highest oxidation current than other modified electrodes, which can be associated with the availability of active sites, surface features, and synergistic effect that efficiently improves electrochemical performance for PMH detection.

### 3.6. Determination of PMH with Cu_3_Mo_2_O_9_/RGO/SPCE Using DPV Technique

**Figure 7:**
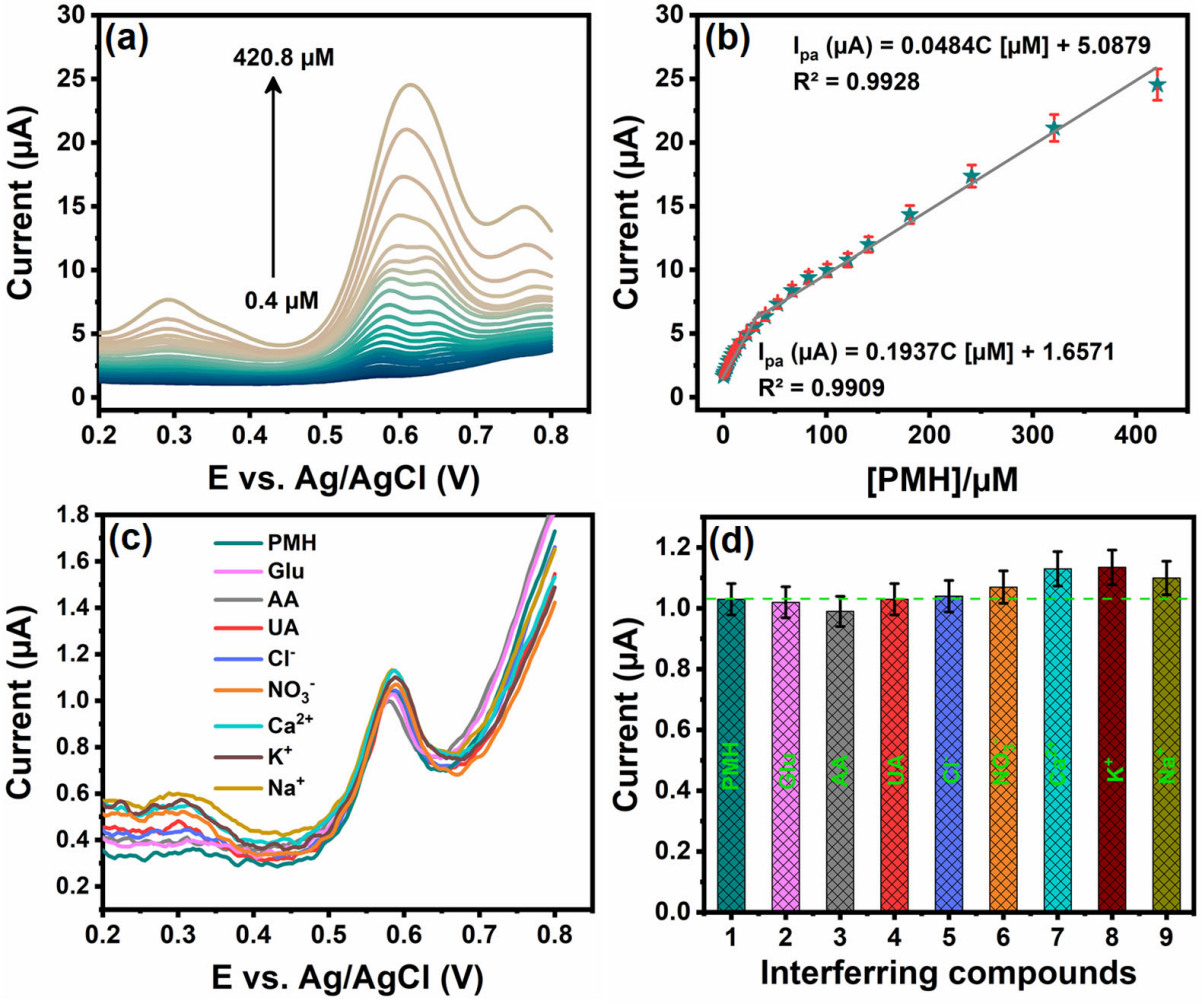
(**a**) DPV of Cu_3_Mo_2_O_9_/RGO/SPCE with diverse PMH concentrations, (**b**) linear curve of oxidation current versus concentrations of PMH, (**c**) interference study of PMH at various biomolecules and metal species, and (**d**) corresponding current response.

To further explore the electrochemical behavior of PMH oxidation at Cu_3_Mo_2_O_9_/RGO/SPCE, the DPV approach was deployed because of its better sensitivity, low detection limit, and high selectivity. Figure 7a shows DPV curves for various PMH quantities in 0.1 M PBS (pH 7) using the Cu_3_Mo_2_O_9_/RGO sensor. The oxidation current rose with an increase in PMH concentrations from 0.4 to 420.8 µM, which indicates that the sensor performed well even at higher additions. The respective oxidation current exhibited two linear curves for PMH concentrations plotted in Figure 7b. The regression equations with correlated coefficients for these curves are I_pa_ (µA) = 0.1937C [µM] + 1.6571 (R^2^ = 0.9909) and I_pa_ (µA) = 0.0484C [µM] + 5.0879 (R^2^ = 0.9928). The limit of detection (LoD) for Cu_3_Mo_2_O_9_/RGO/SPCE was 0.015 µM (estimated using the formula of 3Standared deviation/Slope of the linear curve) [21], suggesting robust electrocatalytic activity. The calculated sensitivity of Cu_3_Mo_2_O_9_/RGO/SPCE for PMH was 0.807 µA µM^−1^ cm^−2^. Furthermore, the designed Cu_3_Mo_2_O_9_/RGO/SPCE sensor was compared to the previously constructed PMH sensors, and the findings are provided in Table 1. Outstandingly, Cu_3_Mo_2_O_9_/RGO/SPCE endowed a relatively low LoD and a broader linear range than that of formerly sensors. The electron transfer capability and electrocatalytic activity of Cu_3_Mo_2_O_9_/RGO/SPCE were enhanced by the synergistic interaction between Cu_3_Mo_2_O_9_ and RGO. Additionally, the proposed sensor is portable, affordable, and simple to construct and identify in the actual samples.

### 3.7. Interference Studies of Cu_3_Mo_2_O_9_/RGO/SPCE

The interference studies of Cu_3_Mo_2_O_9_/RGO/SPCE towards PMH were investigated in the presence of various biomolecules and metal species by the DPV technique. As seen in Figure 7c,d, when 100 µM injections of glucose (Glu), ascorbic acid (AA), uric acid (UA), Cl^−^, NO_3_^−^, Ca^2+^, K^+^, and Na^+^ substances are added to PMH oxidation, the current response was slightly changed (signal recorded below 9.18%), showing that the Cu_3_Mo_2_O_9_/RGO/SPCE composite features good interference ability for PMH oxidation.

**Figure 8:**
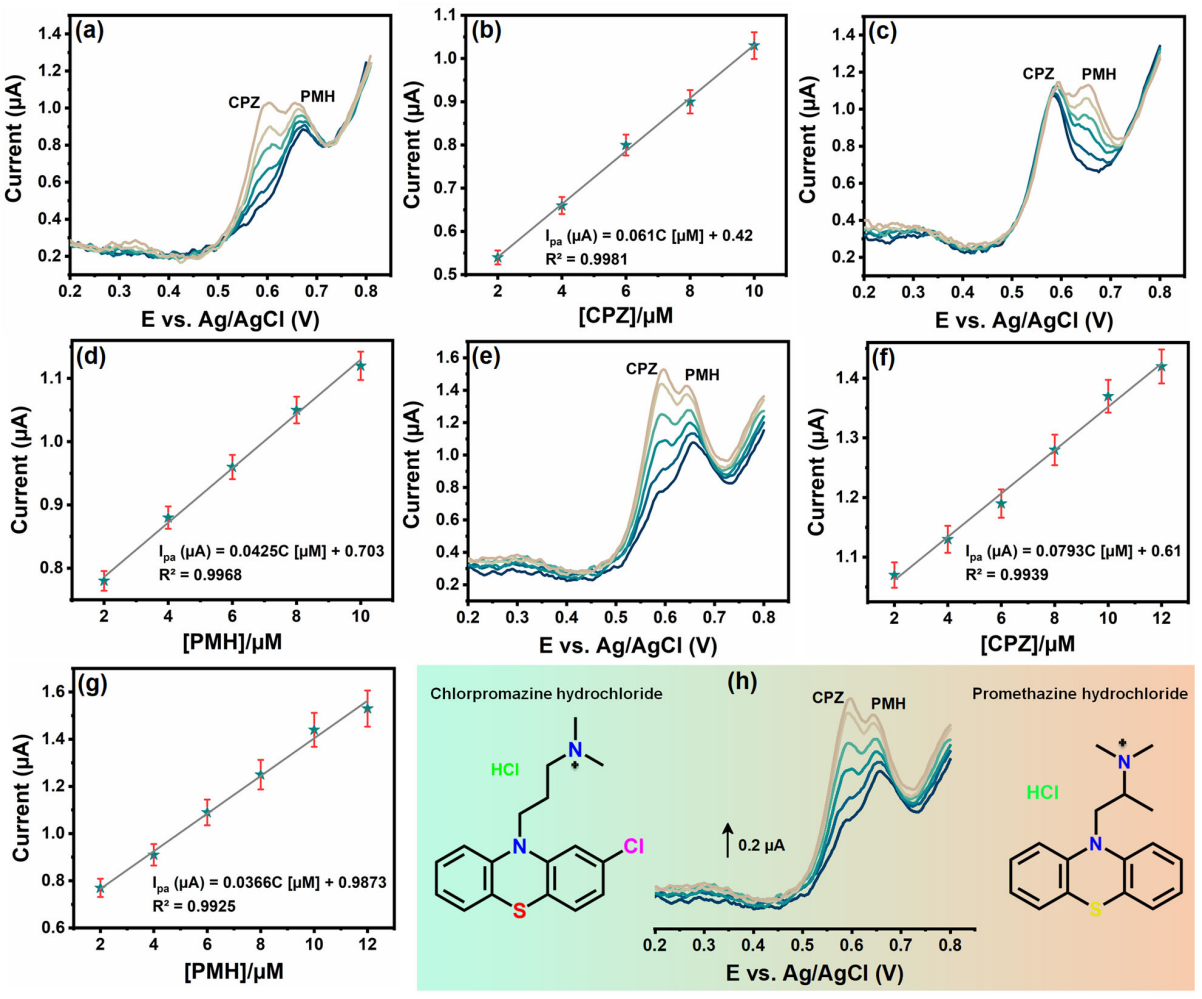
(**a**) DPV response of PMH with the successive additions of CPZ using Cu_3_Mo_2_O_9_/RGO/SPCE, (**c**) DPV response of CPZ with the successive additions of PMH employing Cu_3_Mo_2_O_9_/RGO/SPCE, (**e**) DPV response of PMH and CPZ utilizing Cu_3_Mo_2_O_9_/RGO/SPCE, (**b**,**d**,**f**,**g**) linear plots of oxidation current versus CPZ and PMH concentrations, and (**h**) the chemical structure of PMH and CPZ.

In addition, the DPV was employed to examine the ability of Cu_3_Mo_2_O_9_/RGO/SPCE for the simultaneous monitoring of PMH and other psychotic drug chlorpromazine (CPZ) in 0.1 M PBS (pH 7). Figure 8a,c displays the DPV for various additions of CPZ (2–10 µM) and PMH (2–10 µM) and the fixed addition of PMH (10 µM) and CPZ (10 µM) at Cu_3_Mo_2_O_9_/RGO/SPCE. The oxidation current improved with increasing PMH and CPZ concentrations at the constant PMH and CPZ concentration, which indicates good selectivity for PMH and CPZ detection (Figure 8b,d). Similarly, while increasing CPZ (2–12 µM) and PMH (2–12 µM) concentrations simultaneously (Figure 8e), the oxidation response increased. Figure 8f,g illustrates the linearity of the oxidation current versus the CPZ and PMH concentrations, confirming that this new sensor is highly suitable for the simultaneous assessment of selective antipsychotic drugs.

### 3.8. Cyclic Stability and Reproducibility of the Cu_3_Mo_2_O_9_/RGO/SPCE Sensor

**Figure 9:**
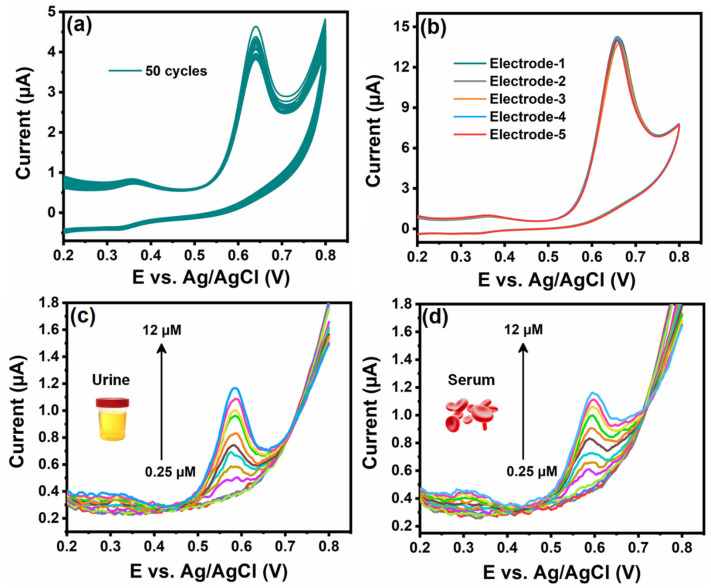
(**a**) CV of Cu_3_Mo_2_O_9_/RGO/SPCE up to 50 continuous cycles for PMH, (**b**) the reproducibility of PMH with five modified Cu_3_Mo_2_O_9_/RGO/SPCE hybrids, and (**c**,**d**) DPV measured at various PMH concentrations in urine and serum samples on Cu_3_Mo_2_O_9_/RGO/SPCE.

Stability and reproducibility are critical aspects that contribute to superior electrochemical performance. The CV evaluated the cyclic stability of Cu_3_Mo_2_O_9_/RGO/SPCE for 50 continuous cycles in PMH (Figure 9a). After 50 cycles, the oxidation current response was around 83.07% of the starting current, representing the satisfactory cycling stability of the sensor for PMH identification. The reproducibility of the Cu_3_Mo_2_O_9_/RGO/SPCE sensor was also investigated with PMH in 0.1 M PBS (pH 7). Almost the same current was seen for five reproduced electrodes, with a relative standard deviation (RSD) of 2.09% (Figure 9b), demonstrating the viability of the Cu_3_Mo_2_O_9_/RGO/SPCE sensor.

### 3.9. Real-Time Monitoring of PMH in Human Samples

Finally, to confirm the practicality and reliability of the Cu_3_Mo_2_O_9_/RGO/SPCE hybrid for the PMH sensor, we examined the real-time monitoring of human (urine and serum) samples using the DPV technique (Figure 9c,d). The real urine and serum samples were added to the buffer solution (pH 7); however, the current response is not shown due to the absence of PMH in the original samples. On the contrary, the oxidation current was detected at 0.57 V upon the known additions of PMH mixed with real samples, revealing high sensitivity. The recovery rate of each sample was measured three times and determined via the standard addition method. Table 2 summarizes the recoveries of PMH ranging from 96.0% to 99.78% in the human urine sample and 94.0% to 99.83% in the human serum sample. The RSD values of PMH were 2.04% and 2.19% for urine and serum samples, respectively. The outcomes proved that the newly discovered Cu_3_Mo_2_O_9_/RGO/SPCE sensor has high accuracy and reliability for sensing PMH in biological fluids, showcasing its potential for practical applications.

## 4. Conclusions

In summary, a sensitive, accurate, and selective electrochemical sensor for PMH detection was designed through the modification of SPCE with Cu_3_Mo_2_O_9_ decorated on an RGO composite. The composition of the hybrid was confirmed via diverse analytical and spectroscopic techniques. The uniform formation of the sheet-like structure of RGO and the sphere-like structure of Cu_3_Mo_2_O_9_ contributed to better electrical conductivity, improved surface properties, and excellent charge transfer ability. As a consequence, compared to Cu_3_Mo_2_O_9_, RGO, and unmodified electrodes, the modified Cu_3_Mo_2_O_9_/RGO/SPCE composite displayed a higher electrochemical signal toward PMH. Moreover, the Cu_3_Mo_2_O_9_/RGO/SPCE PMH sensor demonstrates outstanding electrochemical performance under optimal conditions, featuring an extensive linear range (0.4–420.8 µM), low LoD (0.015 µM), high selectivity, good stability, and reproducibility. The real-time analysis of PMH employing this sensor in urine and serum samples was also proven with acceptable recovery rates. Thus, a facile and effective route was deployed for the first time in order to construct a Cu_3_Mo_2_O_9_/RGO composite for a PMH sensor with improved electrochemical characteristics, highlighting its practicability in biological fluids.

## Figures and Tables

**Table 1:** Comparison study of Cu_3_Mo_2_O_9_/RGO/SPCE to formerly developed materials for the detection of PMH.

Modified Materials	Techniques	Electrolyte/pH	Linear Range (µM)	Detection Limit (µM)	Real Samples	References
MWCNT/SiO_2_/Al_2_O_3_/Nb_2_O_5_/DNA	DPV	PBS/pH 7	20–100	5.9	Tablet	[37]
RGO/GCE	i–t	ABS/pH 5	1.99–1030	0.199	Tablet	[38]
Au/CPE	DPV	PBS/pH 6	2–225	0.065	Tablet and Urine	[32]
Pt/Ni@MWCNT/GCE	FI-i–t	PBS/pH 6	0.1–1000	0.033	Saliva, Beverage, and Urine	[31]
AC/Ag/CPE	DPV	PBS/pH 7	5–40	1.30	Tablet	[39]
bCNT-dsDNA/GCE	DPV	ABS/pH 5	0.1–6	0.023	Tablet	[40]
BSA-O-CQDs	FQ	PBS/pH 8	10–90	0.2	Water	[41]
fMWCNT-PEI/GCE	SWV	BR/pH 2	0.49–5.03	0.231	Tablet	[35]
Pt/ZnO@SPCE	DPV	PBS/pH 7	0.2–116.2	0.0185	Urine	[13]
Cu_3_Mo_2_O_9_/RGO/SPCE	DPV	PBS/pH 7	0.4–420.8	0.015	Urine and Serum	This work

**Table 2:** Real-time monitoring of PMH in human samples on Cu_3_Mo_2_O_9_/RGO/SPCE.

Samples	Added (µM)	Found (µM)	Recovery (%)	RSD (%)
Human urine	H.U-15	Not detected	–	–
	H.U-30	–	–	–
	H.U-50	–	–	–
	0.25	0.24	96.0	2.51
	0.50	0.49	98.0	2.36
	1.0	0.99	99.0	2.18
	2.0	1.97	98.5	2.24
	4.0	3.96	99.0	2.03
	6.0	5.95	99.16	1.87
	8.0	7.98	99.78	1.75
	10.0	9.95	99.5	1.92
	12.0	11.93	99.41	1.50
Human serum	H.S-15	Not detected	–	–
	H.S-30	–	–	–
	H.S-50	–	–	–
	0.25	0.235	94.0	2.78
	0.50	0.48	96.0	2.61
	1.0	0.98	98.0	2.45
	2.0	1.99	99.5	2.37
	4.0	3.94	98.5	2.13
	6.0	5.99	99.83	2.06
	8.0	7.97	99.62	1.94
	10.0	9.93	99.3	1.72
	12.0	11.91	99.25	1.69

## Data Availability

The data will be made available upon request.
